# HCV prevalence and phylogenetic characteristics in a cross‐sectional, community study of young people who inject drugs in New York City: Opportunity for and threats to HCV elimination

**DOI:** 10.1002/hsr2.2211

**Published:** 2024-07-01

**Authors:** Mateu‐Gelabert Pedro, Pratt Seanna, Guarino Honoria, Hallack Renee, Fong Chunki, Eckhardt Ben

**Affiliations:** ^1^ CUNY Graduate School of Public Health and Health Policy New York City New York USA; ^2^ NYS Department of Health Wadsworth Center Albany New York USA; ^3^ NYU School of Medicine New York City New York USA

**Keywords:** dried blood spot testing, hepatitis C virus, people who inject drugs, phylogenetic clustering, prevalence

## Abstract

**Background and Aims:**

In the United States, the opioid epidemic has led many young people who use opioids to initiate injection drug use, putting them at risk for hepatitis C virus (HCV) infection. However, community surveys to monitor HCV prevalence among young people who inject drugs (YPWID) are rare.

**Methods:**

As part of Staying Safe (Ssafe), a trial to evaluate an HCV‐prevention intervention, a community‐recruited sample of 439 young people who use opioids (ages 18−30) in New York City (NYC) were screened from 2018 to 2021. Screening procedures included a brief verbal questionnaire, a visual check for injection marks, onsite urine drug testing, rapid HCV antibody (Ab) testing, and dried blood spot (DBS) collection. DBS specimens were sent to a laboratory for HCV RNA testing and phylogenetic analysis to identify genetic linkages among HCV RNA‐positive specimens. Multivariable logistic regression was used to assess associations between HCV status (Ab and RNA) and demographics and drug use patterns.

**Results:**

Among the 330 participants who reported injecting drugs (past 6 months), 33% (*n* = 110) tested HCV Ab‐positive, 58% of whom (*n* = 64) had HCV RNA‐positive DBS specimens, indicating active infection. In multivariable analysis, visible injection marks (AOR = 3.02; *p* < 0.001), older age (AOR = 1.38; *p* < 0.05), and female gender (AOR = 1.69; *p* = 0.052) were associated with HCV Ab‐positive status. Visible injection marks were also associated with HCV RNA‐positive status (AOR = 5.24; *p* < 0.01). Twenty‐five percent of RNA‐positive specimens (14/57) were genetically linked.

**Conclusion:**

The relatively low prevalence of active infection suggests the potential impact of treatment‐as‐prevention in reducing HCV prevalence among YPWID. Targeted community serosurveys could help identify actively infected YPWID for treatment, thereby reducing HCV incidence and future transmissions.

## 1 INTRODUCTION

Over the past 25 years in the United States, the number of people who engage in nonmedical use of opioids, including prescription opioids, heroin, and, increasingly, fentanyl, has risen markedly, with the largest increases seen in people aged 18−25.^1^ This has led many young people to initiate injection drug use, putting them at risk of exposure to hepatitis C virus (HCV).^2^ Seroprevalence of HCV is high in most US populations of people who inject drugs (PWID), typically ranging from ~40% to 70%, but varies across subpopulations, depending on age and duration of injection drug use, among other factors.^3^, ^4^, ^5^, ^6^, ^7^ National surveillance data from 2021 show that the number of reported acute HCV cases, representing new infections, has doubled since 2014, demonstrating a 129% increase.^8^ The highest rates occurred in those aged 20−39 years, consistent with the age groups most impacted by the opioid crisis. Notably, of cases with risk information, injection drug use was reported among 57% of those cases. Rates of acute HCV among those who inject drugs increased sevenfold among men and threefold among women from 2010 to 2021.^8^

Young people who inject drugs (YPWID) have significantly higher HCV incidence relative to older PWID,^5^, ^9^, ^10^, ^11^, ^12^ though estimates of HCV prevalence are commonly higher among older PWID due to their typically longer duration of injection drug use. A study using 2015 national surveillance data across eight US cities found a 62% antibody (Ab)‐positive seroprevalence among older PWID (>35) compared to 42% among YPWID (≤35).^13^ Most studies have estimated HCV Ab‐positive seroprevalence among US subpopulations of YPWID to range from 33% to 60%,^13^, ^14^, ^15^ with YPWID being most vulnerable to HCV infection in their first 3−5 years of injecting.^9^, ^16^, ^17^ Among a sample of 18−29 year‐old PWID in New York City (NYC assessed in 2014−2016), HCV Ab prevalence was 31%.^18^

Modeling studies have demonstrated that treating active PWID with direct‐acting antivirals (DAA) can have the greatest effect on HCV prevalence reduction through treatment‐as‐prevention.^19^ Due to the higher HCV incidence in young PWID, targeting this subpopulation for treatment has the potential to result in the highest reduction in onward transmission. Identifying subpopulations or clusters of YPWID at higher risk of transmitting HCV could provide public health officials and harm reduction service providers information to identify potential outbreaks and implement targeted interventions.

Phylogenetic analysis allows for the opportunity to examine transmission patterns and identify high‐risk subpopulations or clusters, as demonstrated in prior HIV outbreaks.^20^ Limited literature reports results of phylogenetic analyses examining HCV transmission clusters, specifically among YPWID. The U‐Find‐Out “Partnership Study” conducted in San Francisco enrolled 122 unique young adult injectors from 2006 to 2016. Thirty‐two partnerships were sequenced, and 12 were genetically linked.^21^ In a cohort of older PWID receiving opioid agonist therapy across three clinics in the Bronx, New York (*n* = 138), three HCV transmission linkages were found among RNA‐positive study participants (mean age = 51).^22^ Two studies examined HCV phylogenetics among community samples of PWID, one in Vancouver, Canada, and the other in the United Kingdom.^23^, ^24^ Using data from the Vancouver Injection Drug Users Study, Jacka et al. found that 31% (156/501) of PWID (median age = 36) with the most prevalent HCV genotypes in their sample, were in a pair or cluster of genetically linked HCV cases.^23^

The present study reports the HCV Ab and RNA results of a large community sample of YPWID. In addition, we present molecular epidemiology results assessing how many active infections were genetically related.

## 2 METHODS

### 2.1 Recruitment and procedures

Participants were recruited in NYC between January 2018 and April 2021 as part of the Staying Safe (Ssafe) study, a clinical trial (ClinicalTrials.gov: NCT03418636) that evaluated a behavioral intervention to prevent HCV infection among YPWID. Data for the present study were collected during screening procedures to determine prospective participants' trial eligibility. Before screening procedures, potential participants consented to partake in the screening questionnaire assessing age and English‐language proficiency, providing urine and DBS samples for analysis, and answering sociodemographic and injection‐related questions. The protocol was approved by the CUNY Graduate School of Public Health's Institutional Review Board.

Participants were recruited using systematic sampling techniques outlined by Garfein et al. for YPWID,^25^ including venue‐based, street outreach, and chain‐referral methods. To initiate chain referrals and reach less publicly visible YPWID, recruits were encouraged to distribute study flyers to their peers who use opioids or who know young people who do.

All prospective Ssafe participants who completed screening procedures are included in the overall sample for this analysis, regardless of their trial eligibility or enrollment. Screened individuals who met Ssafe's eligibility criteria (18−29 years old, verified by photo identification; spoke English; reported using opioids nonmedically 12 or more times in the past 30 days; tested opioid‐positive on a urine drug screen; reported injecting drugs at least once in the past 6 months) were enrolled. In addition, for those participants who reported recent drug injections (past ~2 weeks), we respectfully asked if they were willing to show their injection marks in their arms and legs for additional verification of drug injection; however, showing injection marks was not required for enrollment.

A two‐step screening process was used to determine eligibility. The first step involved prescreening prospective participants via self‐report over the phone, ascertaining likely eligibility for the target age range and English‐language proficiency. If these criteria were met, individuals were given an appointment to come to the study site for the full screening process. This second step entailed informed consent for screening, followed by a brief informal interview to verify age and collect information on recent drug use and gender. Consented individuals underwent onsite HIV and HCV rapid Ab testing (OraQuick HCV Rapid Antibody Test and OraQuick Advanced Rapid HIV‐1/2 Antibody Test; OraSure Technologies Inc.) and dried blood spot (DBS). DBS specimens were also collected and sent to an external lab for analysis. Urine drug screening was performed using point‐of‐care test cups (iCup,13 panel) that detected the following agents: buprenorphine, methadone, heroin/opiates, oxycodone, amphetamines, benzodiazepines, cocaine, phencyclidine (PCP), methamphetamines, barbiturates, tricyclic antidepressants, propoxyphene, and marijuana. Fentanyl test strips were introduced during the study as the presence of fentanyl in the NYC illicit drug market increased.^26^ Only those screened after September 2018 were tested for fentanyl. Urine testing was used to verify current opioid use.

### 2.2 DBS laboratory analysis

DBS was sent to the New York State Department of Health Wadsworth Center for HCV RNA testing and Global Hepatitis Outbreak and Surveillance Technology (GHOST) phylogenetic analysis. The laboratory ran qualitative HCV RNA tests on all samples that were HCV Ab‐positive (*n* = 117). Four participants were excluded from the analysis of RNA correlates because the DBS specimen was insufficient to run RNA tests. Qualitative RNA detection from two 6 mm DBS punches was performed with either an in‐house real‐time PCR assay (limit of detection = 500 IU/mL) or the APTIMA HCV Quant Dx assay (Hologic Inc.) using a modified protocol (limit of detection = 250 IU/mL). Both methods were validated and approved by the New York State Department of Health Clinical Laboratory Evaluation Program. Using the Centers for Disease Control and Prevention's protocol version 1.6, “Next Generation Sequencing for HCV HVR1,”^27^ the HCV RNA‐positive specimens deemed viable for phylogenetic analysis (57/69, 83%) were tested using reverse transcription‐polymerase chain reaction to amplify a 264 bp region of the E1/HVR1 junction prone to mutations. Amplification products were sequenced by the Wadsworth Center's Applied Genomics Technology Core using the Miseq system (Illumina Inc.). FastQ sequence files were uploaded into the GHOST portal for analysis to detect variants and identification of transmission links.^28^ Sample sequences differing by <0.037% are considered transmission links. This is the hamming distance‐based threshold value validated by the CDC and cannot be increased but may be decreased.^29^ Linked samples are genetically related but without epidemiological information, source and directionality of infection cannot be concluded.^27^

### 2.3 Variable definitions

HCV Ab‐positive status was determined by a positive result by the onsite rapid Orasure test. For those participants who were HCV Ab‐positive, an HCV RNA test was performed. The variable options used to describe RNA status include: (1) RNA Detected for participants whose DBS specimens resulted in the detection of HCV RNA, and (2) RNA Not Detected for participants who were HCV Ab‐negative or whose DBS specimens had undetectable HCV RNA test results.

For this analysis, injecting at least one time in the past 6 months was coded as (1) yes or (2) no, based on participants self‐reporting having injected any drug(s) during the 6 months before screening. The following variables were assessed as potential correlates of HCV Ab and RNA status among drug injectors (1) age, (2) gender, (3) visible injection marks, and (4) the number of drugs detected in each participant's urine specimen. To reduce the response burden, data on participants' race and ethnicity were not collected during screening interviews, as this information was not pertinent to trial eligibility. The selection of potential correlates for these analyses was informed by prior research by members of our team and the current literature base on factors found to be associated with HCV‐positive status among PWID.

For bivariable and multivariable analyses, variables were recoded as follows. Age was recoded into three categories: (1) 18−21, (2) 22−25, and (3) 26−30 years. The cut‐off for study eligibility was 29 years of age to restrict the sample to young adults, as commonly defined in studies of people who use drugs. However, two participants who were 30 years old at screening were included in this analysis, as they had initially reported being 29 but were determined to be 30 upon photo ID verification. Gender is represented by three categories, (1) male, (2) female, and (3) other (e.g., transgender, nonbinary). Visible injection marks were grouped into three categories (1) yes, seen by staff: which includes those participants who showed visible injection marks to research personnel, (2) yes, self‐reported: which includes participants who self‐reported visible injection marks but did not show them to research personnel, and (3) no: which includes participants who reported not having visible injection marks.

### 2.4 Data analysis

To identify correlates of HCV Ab and HCV RNA positivity among the subsample of YPWID, first crosstabulations of HCV Ab and HCV RNA status were conducted with each variable identified as a potential correlate. Simple logistic regression analyses were then performed with these independent variables to obtain unadjusted odds ratios (95% CI) for HCV Ab status and then again for HCV RNA status. Following this, a multivariable logistic regression analysis was performed, including age, gender, and variables significant at the 0.05 *⍺* level, first for HCV Ab status and then again for HCV RNA status, to obtain adjusted odds ratios (95% CI). All analyses were conducted using SAS (Release 9.4 SAS Institute Inc.).

## 3 RESULTS

Between January 2018 and April 2021, a total of 443 young opioid users were screened. Among them, 1.8% were HIV positive (*n* = 8). Sociodemographic and drug use characteristics and HCV Ab and RNA status of the sample are shown in Table 1. This analysis is based on the 75.2% of participants who reported injecting any drug(s) in the past 6 months (“injectors”; *n* = 330). A third of the participants were HCV Ab‐positive (110/330), and 19.4% were HCV RNA‐positive (64/330). The mean age of injectors was 25.5 (SD = 3.1). Over a quarter (28.8%) were female, and a small percentage (2.7%) reported their gender as other than male or female (e.g., transgender, nonbinary). A majority of participants (78.5%) presented visible injection marks, and 16.4% reported no visible injection marks. Three or more drugs were detected in the urine specimens of 54.8% of participants; 28.5% tested positive for two drugs; 13.9% tested positive for one drug, and 2.7% tested positive for no drugs.

**Table 1:** Demographic and drug use characteristics and HCV serostatus of the total sample of young people reporting drug injection (past 6 months) (*n* = 330).

Characteristic	Participants who injected drugs (past 6 months) *n* (%)
Gender	
Male	226 (68.5)
Female	95 (28.8)
Other (transgender, nonbinary, etc.)	9 (2.7)
Age	
Mean (SD)	25.5 (3.1)
18−21	51 (15.5)
22−25	88 (26.8)
26−30^a^	189 (57.6)
Visible injection marks	
Yes (seen by staff)	259 (78.5)
Yes (self‐report only)	17 (5.2)
No	54 (16.4)
Number of drugs detected in urine	
0	9 (2.7)
1	46 (13.9)
2	94 (28.5)
3 or more	181 (54.8)
HCV antibody status	
Positive	110 (33.3)
Negative	220 (66.7)
HCV RNA status	
Positive	64 (19.4)
Negative	266 (80.6)

Abbreviation: HCV, hepatitis C virus.

^a^ The majority of participants were prescreened over the phone to confirm they were between 18 and 29 years old, but two walk‐ins were found to be 30 during in‐person screening.

In the unadjusted model, HCV Ab positivity was positively correlated with older age (26−30 years) (vs. younger age, 18−21 years) and having visible injection marks that were seen by staff (vs. no visible injection marks). The odds ratios for the unadjusted and adjusted models are displayed in Table 2 *.* In the adjusted model, being in the 26−30 age category (AOR = 1.38; *p* < 0.05) and having visible injection marks (seen by staff) (AOR = 3.02; *p* < 0.01) were significant predictors of HCV Ab‐positive status. Female gender (AOR = 1.69; *p* = 0.052) was a marginally significant predictor of HCV Ab‐positive status.

**Table 2:** Correlates of HCV antibody‐positive serostatus among young people reporting recent injection (past 6 months).^a^

	Total	HCV antibody+	HCV antibody−	OR (95% CI)	*p* Value	AOR (95% CI)	*p* Value
*n* (%)	330 (100.0%)	110 (33.3)	220 (66.7)				
Age		Omnibus test *p*‐value = 0.025					
18−21	51 (15.5%)	12 (10.9%)	39 (17.9%)	Ref	Ref	Ref	Ref
22−25	88 (26.8%)	23 (20.9%)	65 (29.8%)	1.21 (0.54−2.69)	0.642	1.32 (0.58−3.00)	0.506
26−30	189 (57.6%)	75 (68.2%)	114 (52.3%)	1.31 (1.04−1.66)	0.025	1.38 (1.08−1.76)	0.010
Gender		Omnibus test *p*‐value = 0.139					
Male	226 (68.5%)	72 (65.5%)	154 (70.0%)	Ref	Ref	Ref	Ref
Female	95 (28.8%)	37 (33.6%)	58 (26.4%)	1.36 (0.83−2.25)	0.222	1.69 (0.99−2.88)	0.052
Other	9 (2.7%)	1 (0.9%)	8 (3.6%)	0.20 (0.02−1.63)	0.132	0.18 (0.02−1.56)	0.119
Visible injection marks		Omnibus test *p*‐value = 0.002					
No	54 (16.4%)	10 (9.1%)	44 (20.0%)	Ref	Ref	Ref	Ref
Yes, seen by staff	259 (78.5%)	98 (89.1%)	161 (73.2%)	2.68 (1.29−5.56)	0.018	3.02 (1.42−6.43)	0.004
Yes, self‐report	17 (5.2%)	2 (1.8%)	15 (6.8%)	0.59 (0.12−2.99)	0.521	0.59 (0.11−3.08)	0.532
Number of drugs detected in urine		Omnibus test *p*‐value = 0.636					
None	9 (2.7%)	2 (1.8%)	7 (3.2%)	0.73 (0.13−3.96)	0.711	‐‐	‐‐
1	46 (13.9%)	13 (11.8%)	33 (15.0%)	Ref	Ref	‐‐	‐‐
2	94 (28.5%)	30 (27.3%)	64 (29.1%)	1.19 (0.55−2.58)	0.660	‐‐	‐‐
3 or more	181 (54.8%)	65 (59.1%)	116 (52.7%)	1.42 (0.70−2.89)	0.331	‐‐	‐‐

Abbreviation: HCV, hepatitis C virus.

^a^ In the adjusted multivariate model examining HCV‐antibody positive status variables included are age, gender, and visible injection marks. The omnibus test *p*‐value assesses the overall significance of each variable in predicting HCV antibody‐positivity among young people reporting injection in the last 6 months, with a *p* < 0.05 demonstrating significance

Among YPWID who tested HCV Ab‐positive, 58% were RNA‐positive (64/110). In the unadjusted model, HCV RNA positivity was positively correlated with visible injection marks (seen by staff). The odds ratios for HCV RNA‐positive status in the unadjusted and adjusted models are displayed in Table 3 *.* In the adjusted model, having visible injection marks that were seen by staff was significantly correlated with HCV RNA positivity (AOR = 5.24; *p* < 0.01).

**Table 3:** Correlates of HCV RNA‐positive serostatus among young people reporting recent injection (past 6 months).^a^

	Total	HCV RNA detected	No HCV RNA detected	OR (95% CI)	*p* Value	AOR (95% CI)	*p* Value
*n* (%)	330 (100%)	64 (19.4%)	266 (80.6%)				
Age		Omnibus test *p*‐value = 0.578					
18−21	51 (15.5%)	10 (15.6%)	41 (15.5%)	Ref	Ref	Ref	Ref
22−25	88 (26.8%)	14 (21.9%)	74 (28.0%)	0.81 (0.33−1.99)	0.651	0.89 (0.36−2.20)	0.793
26−30	189 (57.6%)	40 (62.5%)	149 (56.4%)	1.05 (0.81−1.36)	0.715	1.08 (0.83−1.41)	0.575
Gender			Omnibus test *p*‐value = 0.139				
Male	226 (68.5%)	44 (68.8%)	182 (70.5%)	Ref	Ref	Ref	Ref
Female	95 (28.8%)	19 (29.7%)	76 (29.5%)	1.03 (0.57−1.89)	0.913	1.183 (0.63−2.21)	0.597
Other	9 (2.7%)	1 (1.6%)	8 (3.0%)	0.50 (0.06−4.25)	0.525	0.46 (0.05−4.02)	0.482
Visible injection marks		Omnibus test *p*‐value = 0.002					
No	54 (16.4%)	3 (4.7%)	51 (19.2%)	Ref	Ref	Ref	Ref
Yes, seen by staff	259 (78.5%)	60 (93.8%)	199 (74.8%)	5.13 (1.54−17.01)	0.008	5.24 (1.57−17.50)	0.007
Yes, self‐reported	17 (5.2%)	1 (1.6%)	16 (6.0%)	1.06 (0.10−10.9)	0.959	1.08 (0.11−11.25)	0.946
Number of drugs detected in urine		Omnibus test *p*‐value = 0.636					
None	9 (2.7%)	1 (1.6%)	8 (3.0%)	0.83 (0.09−7.90)	0.874	‐‐	‐‐
1	46 (13.9%)	6 (9.4%)	40 (15.0%)	Ref	Ref	‐‐	‐‐
2	94 (28.5%)	21 (32.8%)	73 (27.4%)	1.92 (0.72−5.14)	0.195	‐‐	‐‐
3 or more	181 (54.8%)	37 (56.3%)	145 (54.5%)	1.66 (0.65−4.21)	0.290	‐‐	‐‐

Abbreviation: HCV, hepatitis C virus.

^a^ In the adjusted multivariate model examining HCV‐antibody positive status variables included are age, gender, and visible injection marks. The omnibus test *p*‐value assesses the overall significance of each variable in predicting HCV antibody‐positivity among young people reporting injection in the last 6 months, with a *p* < 0.05 demonstrating significance.

**Figure 1:**
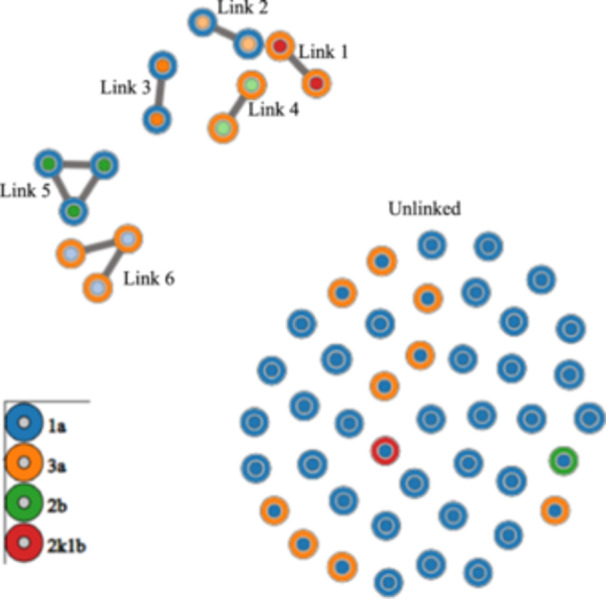
GHOST HCV transmission network graphical representation showing six separate clusters of 14 linked samples and 43 unlinked samples. Each genotype is represented by a colored circle called a node. Lines connecting circles indicate samples differ by a genetic difference ≤0.038. In Link 6, sequences from the middle node are shared independently with the other two nodes which are not linked. HCV, hepatitis C virus.

A majority (57/64; 89%) of HCV RNA‐positive specimens met the criteria for GHOST phylogenetic analysis. HCV strains were distributed among four genotypes: 1a (39/57; 68%); 3a (16/57; 28%); 2b (1/57; 2%); and 2k1b (1/57; 2%). 24.6% (14/57) of HCV RNA‐positive specimens were found to be genetically linked to the specimens of other participants through the identification of six separate transmission links. Four of these links were between two participants, and two of the links were between three participants. All cases are represented in Figure 1. It is worth noting that in Link 6, one participant is genetically similar (middle node) to two others; however, the two others are not linked to each other. This indicates the participants were infected through an unidentified common source case and not through direct transmission amongst this triad.^29^, ^30^

## 4 DISCUSSION

In this large community sample of YPWID in NYC, we found an HCV Ab prevalence of 33.3%, an HCV RNA prevalence of 19.4%, and nearly a quarter of active infections were genetically linked. The HCV Ab prevalence is consistent with the 30.4% prevalence previously reported by our team among a different sample of YPWID in NYC,^18^ and similar to the 34% HCV prevalence reported for YPWID in rural New York.^31^ National surveillance data, however, has estimated HCV Ab prevalence among YPWID to be 42%, and other studies present estimates for this subpopulation ranging from 33% to 60%.^13^, ^14^, ^15^, ^32^ The lower prevalence of HCV Ab positivity in this NYC sample may be related to NYC's long‐sustained harm reduction efforts over the past three decades. Notably, syringe service programs (SSPs) have been legally operating and publicly funded in NYC since 1992,^18^ whereas many other areas of the US only gained explicit authorization to operate these programs within the last 8 years. As recently as 2018, nearly half of all SSPs in the United States were located in five states (California, New Mexico, North Carolina, New York, and Kentucky), demonstrating the country's uneven geographical distribution of services that are critical to preventing the spread of HCV.^33^

Of the participants who were HCV Ab‐positive, 58% were HCV RNA‐positive, indicating active infection. This suggests that up to 42% of this sample of YPWID may have cleared their infection spontaneously or possibly through treatment. A cross‐sectional study by members of our team (*n* = 105) found that only 6% of a sample of YPWID assessed in NYC in 2014−2016 had initiated HCV treatment.^34^ This, along with other research showing that YPWID faces multiple barriers to and has low uptake rates of DAA treatment,^35^ suggests that a majority of Ab‐positive/RNA‐negative participants in our study may have spontaneously cleared their infection. However, it is possible the number of YPWID who have engaged in HCV treatment could have increased in the past few years with better availability of DAAs. The rate of active infection among Ab‐positive participants (58%) is lower than the 76% reported for a community sample of HCV Ab‐positive PWID in Vancouver and the 64% reported in a US national sample of PWID, though these studies report rates that also include older PWID.^23^, ^36^ These results suggest that YPWID who are exposed to HCV may be more likely to clear their infection as compared to older PWID. This aligns with the results of a systematic review and meta‐analysis, which found that, among PWID, spontaneous clearance is lower among older individuals and may be related to age‐related differences in immune function.^37^, ^38^

A study reporting on HCV chronic infection among YPWID, the UFO‐3 prospective cohort study in San Francisco, found that among those recently HCV exposed, 21% spontaneously cleared their infection, and 79% became chronically infected (or were undetermined).^14^ This is a far higher rate of chronic infection than the rate of active infection observed in our sample (58%), suggesting that further research is needed to determine why such apparent variance in HCV natural clearance was found between YPWID in two US urban settings.

Nearly 25% of RNA‐positive samples in our study were determined to be phylogenetically linked, suggesting a fairly high amount of intra‐group transmission among YPWID. Although this may in part be related to our use of chain‐referral as one of the study's recruitment methods, the high rate of phylogenetic linkages may also indicate that the injection networks of YPWID in NYC are substantially interconnected, facilitating HCV transmission in this subpopulation. It is possible that, given the history of high HCV prevalence among older PWID, phylogenetic linkages could occur between older and younger PWID. However, prior research has found that YPWID's injection networks are largely comprised of individuals of a similar age.^39^, ^40^ In contrast, a study among older PWID in treatment centers in the Bronx, NYC, phylogenetic linkages were found among only 2% of the sample,^22^ perhaps indicating older PWID have wider networks and hence a wider pool of source HCV infections in comparison to YPWID.

The relatively low rate of active infection among this community sample of YPWID (19.4%), the suggestion of a potentially high rate of natural clearance, and the high rate of phylogenetic links among active infections suggest the possibility of HCV micro‐elimination among YPWID in NYC. For example, harm reduction services could engage in HCV RNA testing of all YPWID and provide onsite treatment to those who are RNA‐positive. This initiative would entail offering low‐threshold, non‐stigmatizing HCV treatment to any young person who is HCV RNA‐positive at sites where infected individuals may already attend, such as SSPs.^41^ It is important to note that a significant barrier to this strategy is the relatively low rate of SSP use among YPWID compared to older PWID, requiring further innovation in strategies to effectively reach and engage YPWID.^42^, ^43^, ^44^ As suggested by Palmer et al.,^45^ future studies could incentivize referrals for engagement in testing and treatment. Despite this challenge, recent studies published by our group indicate that YPWID can successfully be treated at SSPs immediately after becoming aware of their active infection.^41^ HCV treatment‐as‐prevention would not only cure the infections of young individuals but could also drastically reduce the baseline prevalence in YPWID, thereby preventing further infections and potentially eliminating HCV in this subpopulation.

Significant predictors of HCV Ab positivity included older age (age 26−30) and female gender. Those of older age being significantly more likely to be HCV Ab positive is consistent within the literature, as older individuals are more likely to have a longer history of injection, and thus more opportunities for HCV exposure.^13^, ^18^ The findings for gender are also consistent with the literature, with females being marginally more likely to be HCV Ab positive compared to males. While some studies have demonstrated that women may have a higher likelihood of spontaneous clearance of HCV, women who inject drugs are simultaneously at a higher risk of HCV exposure. This could be due to women being more likely to be injected by others and to engage in receptive sharing of syringes and other injection equipment, especially with male sex partners. Other gender‐related factors that may explain women's increased odds of HCV Ab positivity include threats or experiences of violence and complex power dynamics in relationships that inhibit women's ability to implement safer drug use practices.^46^, ^47^, ^48^, ^49^

In the multivariable analysis, only one hypothesized predictor, the presence of visible injection marks confirmed by research staff, was significantly associated with both HCV Ab and RNA positivity. Participants with visible injection marks had a three times greater likelihood of being HCV Ab‐positive and over five times greater likelihood of being HCV RNA‐positive. Visible injection marks may function as a marker of injection severity (e.g., frequent injection, polysubstance use, reuse, or sharing of injection equipment) that could also present repeated opportunities for exposure to HCV. To our knowledge, no other study has reported on visible injection marks as a predictor of HCV exposure and active infection. This finding could be of importance for harm reduction field operations and prevention efforts, given that visible injection marks are easily identifiable through observation and do not require biotesting. When identified in a non‐stigmatizing way, ideally in the context of an existing supportive relationship, this visible marker could help identify those most at risk of HCV for testing and in need of treatment. Other research identifies multiple risk factors (e.g., sharing of injection equipment, homelessness) related to HCV positivity, though many of these require a behavioral survey to detect.

This study has strengths and limitations to note. To our knowledge, this is the first study in the United States that reports HCV Ab status, HCV RNA status, and HCV phylogenetic links in a large community sample of young PWID. While DBS specimens have proven utility in molecular testing,^50^, ^51^ and are less invasive than venous blood draws, using DBS raises the limit of detection of HCV RNA tests and the ability to sequence when compared to “gold‐standard” plasma samples. This is due to a vastly lower input volume when testing. However, if samples do have enough RNA to sequence, HCV diversity between plasma‐derived and DBS‐derived specimens produces sequences that are highly concordant.^52^ It should also be noted that RNA positivity could be underestimated due to fluctuating viremia levels that could occur during acute infection or from false negative Ab test results. Transitioning to the Aptima assay, which had a lower limit of detection than the real‐time PCR assay, helped reduce the likelihood of false negatives in viremic patients. Additionally, an HIV test was performed, and relatively few (*n* = 8) were HIV positive, reducing the likelihood of false negatives due to HIV positivity.

The study is also limited by the use of convenience sampling methods which limits generalizability and potentially overestimates the level of genetically linked HCV infections since participants may have been more likely to refer those they injected with to the study. However, given that nonmedical use of opioids and heroin use are illegal and stigmatizing activities, random sampling is not feasible and would not yield the large community sample we were able to recruit using a diverse set of sampling techniques. While self‐reported recent injection drug use may be under‐reported due to social desirability bias, such risk is likely to be minimal among this sample given the willingness of participants to be screened for a drug use‐related study and to provide urine specimens to assess recent drug use. Due to the cross‐sectional nature of the data reported here, we cannot determine the temporal associations between variables. However, the associations between visible injection marks and HCV infection have strong face validity in that injection marks are caused by repeated drug injection, and injecting with non‐sterile injection equipment may promote the development of injection marks. Further research is needed to explore if visible injection marks are, in fact, associated with active HCV infection among PWID in other localities.

## AUTHOR CONTRIBUTIONS

**Mateu‐Gelabert Pedro**: Conceptualization; data curation; formal analysis; funding acquisition; investigation; methodology; project administration; supervision; writing—original draft; writing—review and editing. **Pratt Seanna**: Data curation; investigation; writing—original draft; writing—review and editing. **Guarino Honoria**: Conceptualization; data curation; funding acquisition; investigation; methodology; project administration; writing—review and editing. **Hallack Renee**: Methodology; resources; writing—review and editing. **Fong Chunki**: Data curation; formal analysis; methodology; writing—review and editing. **Eckhardt Ben**: Writing—review and editing. All authors have read and approved the final version of the manuscript.

## CONFLICT OF INTEREST STATEMENT

The authors declare no conflict of interest.

## TRANSPARENCY STATEMENT

The lead author Pratt Seanna affirms that this manuscript is an honest, accurate, and transparent account of the study being reported; that no important aspects of the study have been omitted; and that any discrepancies from the study as planned (and, if relevant, registered) have been explained.

## Data Availability

The data that support the findings of this study are available on request from the corresponding author [S. P.]. The data are not publicly available due to the data containing information that could compromise the privacy of research participants. Pedro Mateu‐Gelabert had full access to all of the data in this study and takes complete responsibility for the integrity of the data and the accuracy of the data analysis.

## References

[1] Jones CM , Logan J , Gladden MR , Bohm M . Vital signs: demographic and substance use trends among heroin users—United States, 2002–2013 [Internet]. 2015;64:719‐725. https://www.cdc.gov/mmwr/preview/mmwrhtml/mm6426a3.htm PMC4584844 26158353

[2] Guarino H , Mateu‐Gelabert P , Teubl J , Goodbody E . Young adults' opioid use trajectories: from nonmedical prescription opioid use to heroin, drug injection, drug treatment and overdose. Addict Behav. 2018;86:118‐123. 29747875 10.1016/j.addbeh.2018.04.017 PMC6377245

[3] Jordan AE , Des Jarlais DC , Arasteh K , McKnight C , Nash D , Perlman DC . Incidence and prevalence of hepatitis C virus infection among persons who inject drugs in New York city: 2006‐2013. Drug Alcohol Depend. 2015;152:194‐200. 25891230 10.1016/j.drugalcdep.2015.03.039 PMC4458155

[4] Klevens RM , Hu DJ , Jiles R , Holmberg SD . Evolving epidemiology of hepatitis C virus in the United States. Clin Infect Dis. 2012;55(suppl 1):S3‐S9. 22715211 10.1093/cid/cis393 PMC5774980

[5] Hagan H , Pouget ER , Williams IT , et al. Attribution of hepatitis C virus seroconversion risk in young injection drug users in 5 US cities. J Infect Dis. 2010;201(3):378‐385. 20053137 10.1086/649783

[6] Amon JJ , Garfein RS , Ahdieh‐Grant L , et al. Prevalence of hepatitis C virus infection among injection drug users in the United States, 1994‐2004. Clin Infect Dis. 2008;46(12):1852‐1858. 18462109 10.1086/588297

[7] Armstrong GL , Wasley A , Simard EP , McQuillan GM , Kuhnert WL , Alter MJ . The prevalence of hepatitis C virus infection in the United States, 1999 through 2002. Ann Intern Med. 2006;144(10):705‐714. 16702586 10.7326/0003-4819-144-10-200605160-00004

[8] 2021 Hepatitis C | Viral hepatitis surveillance report | CDC [Internet] . 2023 Accessed April 16, 2024. https://www.cdc.gov/hepatitis/statistics/2021surveillance/hepatitis-c.htm

[9] Becker Buxton M , Vlahov D , Strathdee SA , et al. Association between injection practices and duration of injection among recently initiated injection drug users. Drug Alcohol Depend. 2004;75(2):177‐183. 15276223 10.1016/j.drugalcdep.2004.01.014

[10] Evans JL , Hahn JA , Lum PJ , Stein ES , Page K . Predictors of injection drug use cessation and relapse in a prospective cohort of young injection drug users in San Francisco, CA (UFO Study). Drug Alcohol Depend. 2009;101(3):152‐157. 19181458 10.1016/j.drugalcdep.2008.12.007 PMC2692897

[11] Des Jarlais DC , Arasteh K , McKnight C , et al. HIV infection during limited versus combined HIV prevention programs for IDUs in New York city: the importance of transmission behaviors. Drug Alcohol Depend. 2010;109(0):154‐160. 20163922 10.1016/j.drugalcdep.2009.12.028 PMC4447191

[12] Korthuis PT , Feaster DJ , Gomez ZL , et al. Injection behaviors among injection drug users in treatment: the role of hepatitis C awareness. Addict Behav. 2012;37(4):552‐555. 22209655 10.1016/j.addbeh.2011.12.001 PMC3288438

[13] Abara WE , Trujillo L , Broz D , et al. Age‐related differences in past or present hepatitis C virus infection among people who inject drugs: national human immunodeficiency virus behavioral surveillance, 8 US cities, 2015. J Infect Dis. 2019;220(3):377‐385. 30915477 10.1093/infdis/jiz142 PMC11111175

[14] Page K , Hahn JA , Evans J , et al. Acute hepatitis C virus infection in young adult injection drug users: a prospective study of incident infection, resolution, and reinfection. J Infect Dis. 2009;200(8):1216‐1226. 19764883 10.1086/605947 PMC2821203

[15] Miller CL , Wood E , Spittal PM , et al. The future face of coinfection: prevalence and incidence of HIV and hepatitis C virus coinfection among young injection drug users. JAIDS J Acq Imm Defic Syndromes. 2004;36(2):743‐749. 10.1097/00126334-200406010-00012 15167294

[16] Hagan H , Pouget ER , Des Jarlais DC , Lelutiu‐Weinberger C . Meta‐regression of hepatitis C virus infection in relation to time since onset of illicit drug injection: the influence of time and place. Am J Epidemiol. 2008;168(10):1099‐1109. 18849303 10.1093/aje/kwn237 PMC2727245

[17] Grebely J , Dore GJ . Prevention of hepatitis C virus in injecting drug users: a narrow window of opportunity. J Infect Dis. 2011;203(5):571‐574. 21282190 10.1093/infdis/jiq111 PMC3072734

[18] Mateu‐Gelabert P , Sabounchi NS , Guarino H , et al. Hepatitis C virus risk among young people who inject drugs. Front Public Health. 2022;10:835836. 35968435 10.3389/fpubh.2022.835836 PMC9372473

[19] Zelenev A , Li J , Mazhnaya A , Basu S , Altice FL . Hepatitis C virus treatment as prevention in an extended network of people who inject drugs in the USA: a modelling study. Lancet Infect Dis. 2018;18(2):215‐224. 29153265 10.1016/S1473-3099(17)30676-X PMC5860640

[20] Peters PJ , Pontones P , Hoover KW , et al. HIV infection linked to injection use of oxymorphone in Indiana, 2014–2015. N Engl J Med. 2016;375(3):229‐239. 27468059 10.1056/NEJMoa1515195

[21] Tully DC , Hahn JA , Bean DJ , et al. Identification of genetically related HCV infections among self‐described injecting partnerships. Clin Infect Dis. 2022;74(6):993‐1003. 34448809 10.1093/cid/ciab596 PMC8946742

[22] Akiyama MJ , Lipsey D , Ganova‐Raeva L , et al. A phylogenetic analysis of hepatitis C virus transmission, relapse, and reinfection among people who inject drugs receiving opioid agonist therapy. J Infect Dis. 2020;222(3):488‐498. 32150621 10.1093/infdis/jiaa100 PMC7336560

[23] Jacka B , Applegate T , Krajden M , et al. Phylogenetic clustering of hepatitis C virus among people who inject drugs in Vancouver, Canada. Hepatology. 2014;60(5):1571‐1580. 25042607 10.1002/hep.27310 PMC4211947

[24] Hope VD , Hickman M , Ngui SL , et al. Measuring the incidence, prevalence and genetic relatedness of hepatitis C infections among a community recruited sample of injecting drug users, using dried blood spots. J Viral Hepatitis. 2011;18(4):262‐270. 10.1111/j.1365-2893.2010.01297.x 20456636

[25] Garfein RS , Swartzendruber A , Ouellet LJ , et al. Methods to recruit and retain a cohort of young‐adult injection drug users for the Third Collaborative Injection Drug Users Study/Drug Users Intervention Trial (CIDUS III/DUIT). Drug Alcohol Depend. 2007;91(suppl 1):S4‐S17. 17582705 10.1016/j.drugalcdep.2007.05.007

[26] McKnight C , Des Jarlais DC . Being “hooked up” during a sharp increase in the availability of illicitly manufactured fentanyl: adaptations of drug using practices among people who use drugs (PWUD) in New York city. International J Drug Policy. 2018;60:82‐88. 10.1016/j.drugpo.2018.08.004 PMC6457118 30176422

[27] Longmire AG , Sims S , Rytsareva I , et al. GHOST: global hepatitis outbreak and surveillance technology. BMC Genomics. 2017;18(10):916. 29244005 10.1186/s12864-017-4268-3 PMC5731493

[28] Kerr RJS , Player G , Fiscus SA , Nelson JAE . Qualitative human immunodeficiency virus RNA analysis of dried blood spots for diagnosis of infections in infants. J Clin Microbiol. 2009;47(1):220‐222. 19005148 10.1128/JCM.01521-08 PMC2620881

[29] Campo DS , Xia GL , Dimitrova Z , et al. Accurate genetic detection of hepatitis C virus transmissions in outbreak settings. J Infect Dis. 2016;213(6):957‐965. 26582955 10.1093/infdis/jiv542 PMC5119477

[30] Akiyama M , Khudyakov Y , Ramachandran S , et al. Country‐wide hepatitis C virus transmission networks among people who inject drugs in Kenya [Internet]; 2024. Accessed April 23, 2024. https://papers.ssrn.com/abstract=4700104 10.1016/j.ijid.2024.107215 PMC11531246 39182826

[31] Zibbell JE , Hart‐Malloy R , Barry J , Fan L , Flanigan C . Risk factors for HCV infection among young adults in rural New York who inject prescription opioid analgesics. Am J Public Health. 2014;104(11):2226‐2232. 25211717 10.2105/AJPH.2014.302142 PMC4202941

[32] Wagner K , Zhong Y , Teshale E , et al. Hepatitis C virus infection and polysubstance use among young adult people who inject drugs in a rural county of New Mexico. Drug Alcohol Depend. 2021;220:108527. 33465605 10.1016/j.drugalcdep.2021.108527 PMC7889731

[33] Fernández‐Viña MH , Prood NE , Hepolsheimer A , Waimberg J , Burris S . State laws governing syringe services programs and participant syringe possession, 2014‐2019. Public Health Rep. 2020;135(1):128S‐137S. 32735195 10.1177/0033354920921817 PMC7407055

[34] Kapadia SN , Katzman C , Fong C , Eckhardt BJ , Guarino H , Mateu‐Gelabert P . Hepatitis C testing and treatment uptake among young people who use opioids in New York city: a cross‐sectional study. J Viral Hepatitis. 2021;28(2):326‐333. 10.1111/jvh.13437 PMC8207521 33141503

[35] Morris MD , Mirzazadeh A , Evans JL , et al. Treatment cascade for hepatitis C virus in young adult people who inject drugs in San Francisco: low number treated. Drug Alcohol Depend. 2019;198:133‐135. 30921649 10.1016/j.drugalcdep.2019.02.008 PMC6482851

[36] Assoumou SA , Wang J , Nolen S , et al. HCV testing and treatment in a national sample of US federally qualified health centers during the opioid epidemic. J Gen Intern Med. 2020;35(5):1477‐1483. 32133577 10.1007/s11606-020-05701-9 PMC7210368

[37] Smith DJ , Jordan AE , Frank M , Hagan H . Spontaneous viral clearance of hepatitis C virus (HCV) infection among people who inject drugs (PWID) and HIV‐positive men who have sex with men (HIV+ MSM): a systematic review and meta‐analysis. BMC Infect Dis. 2016;16(1):471. 27595855 10.1186/s12879-016-1807-5 PMC5011802

[38] Grebely J , Prins M , Hellard M , et al. Hepatitis C virus clearance, reinfection, and persistence, with insights from studies of injecting drug users: towards a vaccine. Lancet Infect Dis. 2012;12(5):408‐414. 22541630 10.1016/S1473-3099(12)70010-5 PMC3608418

[39] Page K , Evans JL , Hahn JA , Vickerman P , Shiboski S , Morris MD . HCV incidence is associated with injecting partner age and HCV serostatus mixing in young adults who inject drugs in San Francisco. PLoS One. 2019;14(12):e0226166. 31821365 10.1371/journal.pone.0226166 PMC6903751

[40] Mateu‐Gelabert P , Guarino H , Quinn K , et al. Young drug users: a vulnerable population and an underutilized resource in HIV/HCV prevention. Curr HIV/AIDS Rep. 2018;15(4):324‐335. 29931468 10.1007/s11904-018-0406-z PMC6309604

[41] Eckhardt B , Mateu‐Gelabert P , Aponte‐Melendez Y , et al. Accessible hepatitis C care for people who inject drugs: a randomized clinical trial. JAMA Internal Med. 2022;182(5):494‐502. 35285851 10.1001/jamainternmed.2022.0170 PMC8922207

[42] NYC Health . Epi data brief: syringe service programs in New York City. NYCDOHMH; 2019.

[43] Calvo M , MacFarlane J , Zaccaro H , et al. Young people who use drugs engaged in harm reduction programs in New York city: overdose and other risks. Drug Alcohol Depend. 2017;178:106‐114. 28645060 10.1016/j.drugalcdep.2017.04.032

[44] Ohringer AR , Serota DP , McLean RL , Stockman LJ , Watt JP . Disparities in risk perception and low harm reduction services awareness, access, and utilization among young people with newly reported hepatitis C infections in California, 2018. BMC Public Health. 2021;21(1):1435. 34289822 10.1186/s12889-021-11492-3 PMC8296725

[45] Palmer A , Chan K , Gold J , et al. A modelling analysis of financial incentives for hepatitis C testing and treatment uptake delivered through a community‐based testing campaign. J Viral Hepatitis. 2021;28:1624‐1634. 10.1111/jvh.13596 34415639

[46] Iversen J , Page K , Madden A , Maher L . HIV, HCV and health‐related harms among women who inject drugs: implications for prevention and treatment. JAIDS J Acq Imm Defic Syndromes. 2015;69(01):S176‐S181. 10.1097/QAI.0000000000000659 PMC4505917 25978485

[47] Tracy D , Hahn JA , Lewis C , et al. Higher risk of incident hepatitis C virus among young women who inject drugs compared with young men in association with sexual relationships: a prospective analysis from the UFO Study cohort. BMJ Open. 2014;4(5):e004988. 10.1136/bmjopen-2014-004988 PMC4039809 24875490

[48] Evans JL , Hahn JA , Page‐Shafer K , et al. Gender differences in sexual and injection risk behavior among active young injection drug users in San Francisco (the UFO Study). J Urban Health: Bulletin New York Acad Med. 2003;80(1):137‐146. 10.1093/jurban/jtg137 PMC3456106 12612103

[49] Artenie A , Stone J , Fraser H , et al. Incidence of HIV and hepatitis C virus among people who inject drugs, and associations with age and sex or gender: a global systematic review and meta‐analysis. Lancet Gastroenterol Hepatol. 2023;8(6):533‐552. 36996853 10.1016/S2468-1253(23)00018-3 PMC10817215

[50] Catlett B , Bajis S , Starr M , et al. Evaluation of the Aptima HCV Quant Dx assay for hepatitis C virus RNA detection from fingerstick capillary dried blood spot and venepuncture‐collected samples. J Infect Dis. 2021;223(5):818‐826. 32710758 10.1093/infdis/jiaa442

[51] Carty PG , McCarthy M , O'Neill SM , et al. Laboratory‐based testing for hepatitis C infection using dried blood spot samples: a systematic review and meta‐analysis of diagnostic accuracy. Rev Med Virol. 2022;32(4):e2320. 34957630 10.1002/rmv.2320

[52] Tully DC , Power KA , Sarette J , et al. Validation of dried blood spots for capturing hepatitis C virus diversity for genomic surveillance. J Viral Hepatitis. 2024;31(5):266‐270. 10.1111/jvh.13924 PMC11023755 38366329

